# Mitochondria as Targets for Endothelial Protection in COVID-19

**DOI:** 10.3389/fphys.2020.606170

**Published:** 2020-11-19

**Authors:** Boris V. Chernyak, Ekaterina N. Popova, Ludmila A. Zinovkina, Konstantin G. Lyamzaev, Roman A. Zinovkin

**Affiliations:** ^1^A.N. Belozersky Institute of Physico-Chemical Biology, M.V. Lomonosov Moscow State University, Moscow, Russia; ^2^Faculty of Bioengineering and Bioinformatics, M.V. Lomonosov Moscow State University, Moscow, Russia; ^3^Institute of Molecular Medicine, Sechenov First Moscow State Medical University, Moscow, Russia

**Keywords:** COVID-19, endothelium, mitochondria, reactive oxygen species, mitochondria-targeted antioxidants

## Introduction

The endothelium serves as one of the main targets of the SARS-CoV-2 virus, and endothelial dysfunction largely determines the pathogenesis and clinical outcome in COVID-19 (Teuwen et al., 2020). Pathophysiological studies demonstrated inflammatory activation of the endothelium, destruction of intercellular contacts, and disruption of contacts with the basement membrane in COVID-19 (Ackermann et al., 2020). Dysfunction of the endothelium was one of the reasons for thrombosis, both of the pulmonary capillaries and deep veins. At the same time, there was a significant stimulation of angiogenesis (Teuwen et al., 2020) caused by damage to the endothelium and hypoxia in the affected areas of the lung. Signs of viral infection of endothelial cells were found not only in the vessels of the lungs, but also in the heart and other organs, and were also confirmed *in vitro* using a model of vascular organoids (Monteil et al., 2020).

Mitochondria in endothelial cells do not play a large role in energy metabolism but they determine many cellular responses by controlling the most important signaling pathways. It can be assumed that drugs targeting mitochondria could serve as an important tool for protecting the endothelium in severe forms of COVID-19. The interactomes of SARS-CoV-2 proteins (Gordon et al., 2020; Guzzi et al., 2020; Srinivasan et al., 2020) and transcriptome analysis (Gardinassi et al., 2020) predicted that mitochondria could serve as a direct target for viral proteins expressed in the cell.

## Possible Role of Mitochondria in Endothelial Response to COVID-19 Infection

### Interferon Signaling and Mitochondria

Type I interferon (IFN-I) activation is a central component of cellular antiviral defense (Beck and Aksentijevich, 2020). Viral RNA is recognized by membrane-bound TLR3 and by soluble receptors RIG-I and MDA5. Downstream IFN-I signaling involves mitochondrial adapters MAVS/TOM70/TRAF3/TRAF6 attached to the outer mitochondrial membrane at the contact sites with the endoplasmic reticulum (the so-called mitochondria-associated membranes, MAMs). IFN-I signaling and various anti-IFN strategies of positive-sense RNA viruses including coronaviruses have recently been extensively reviewed (Gatti et al., 2020).

**Figure 1:**
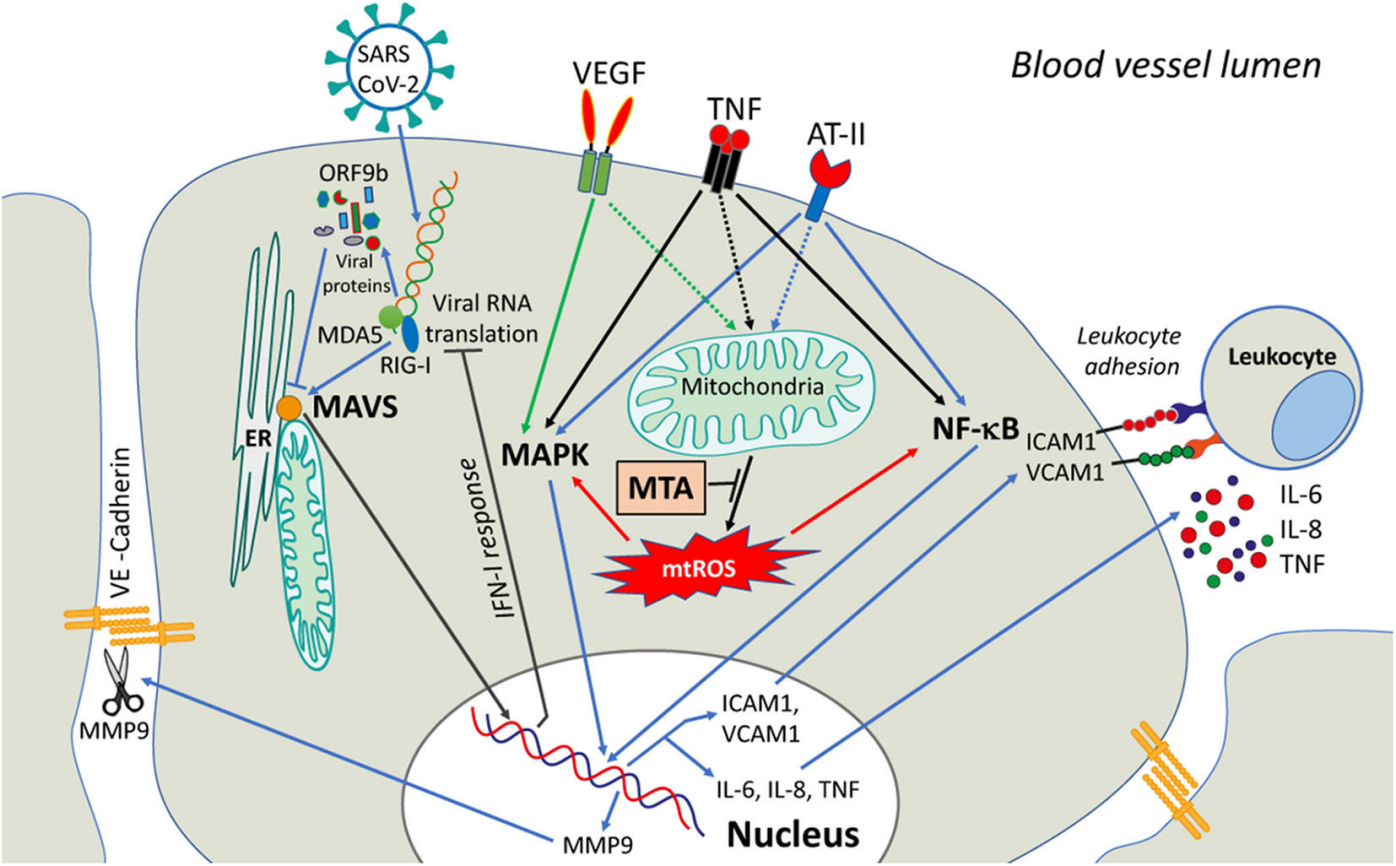
A possible role of mitochondria in signaling induced in endothelial cells by COVID-19 infection. SARS-CoV-2 viral infection causes the release of Vascular Endothelial Growth Factor (VEGF), angiotensin-II (ATII), and circulatory pro-inflammatory cytokines (like TNF). The activation of downstream signaling pathways involves NF-κB and MAPK and upregulates the expression of MMP9 protease, adhesion molecules (ICAM1, VCAM1, etc.), and pro-inflammatory cytokines. MMP9 degrades VE-cadherin and disrupts adhesive contacts, increasing vascular permeability. The adhesion molecules stimulate the adhesion of activated leukocytes and interfere with the assembly of the adhesive contacts (not shown). The activation of both NF-κB and MAPK signaling is dependent on mtROS that can be overproduced by dysfunctional mitochondria. MtROS can directly affect the actin cytoskeleton interacting with adhesive contacts via beta-catenin adapter proteins, further promoting endothelial permeability (not shown). Scavenging of mtROS with mitochondria-targeted antioxidants (MTA) may protect the endothelium from excessive inflammation caused by SARS-CoV-2.

The only experimental study of the interactions of SARS-CoV-2 proteins with mitochondria (Jiang et al., 2020), has shown that ORF9b co-localizes to mitochondria and interacts with the outer mitochondrial membrane protein TOM70 in full agreement with the interactome prediction (Gordon et al., 2020). Importantly, this interaction suppresses IFN-I response and overexpression of TOM70 abolished this inhibition. The inhibition of IFN-I signaling by the homologous protein of SARS-CoV-1 was reported earlier (Shi et al., 2014). A scheme depicting IFN-I inhibition by SARS-CoV-2 is shown in Figure 1.

Hypothetically, ORF9b may deactivate MAVS by interfering with the TOM70-HSP90 interaction (Liu et al., 2010) or by activating the proteolytic degradation of MAVS, TRAF3, and TRAF6 (Shi et al., 2014). Alternatively, ORF9b may interfere with the formation of MAMs by affecting mitochondrial dynamics. SARS-CoV-1 ORF9b induced mitochondrial elongation by activating proteasome degradation of the dynamin-like protein (DRP1), a GTPase responsible for mitochondrial fragmentation (Shi et al., 2014). The more common viral strategies are mediated by the induction of mitochondrial fragmentation, but the Dengue and Zika viruses induced mitochondrial elongation associated with the formation of disorganized (convoluted) ER membranes (Chatel-Chaix et al., 2016). Both scenarios can prevent the assembly of MAM and the activation of MAVS.

It was predicted that some other SARS-CoV-2 proteins interfere with IFN-I signaling and interact with the mitochondrial respiratory chain, the protein import machinery, and ribosomes (Gatti et al., 2020; Gordon et al., 2020). These interactions may promote viral replication by modulating mitochondrial metabolism and/or preventing the early induction of apoptosis (Gatti et al., 2020). At the later stages of infection, these proteins may stimulate cell death, helping viral dissemination. These effects are awaiting experimental investigation.

### Excessive Inflammation and Mitochondria

The interaction of the endothelium of the pulmonary capillaries with the SARS-CoV-2 virus can occur in the early stages of the disease, given the possibility of the virus entering the bloodstream without destroying the cells of the alveolar epithelium and the proximity of the capillary endothelium. At later stages, the virus can induce an endothelial response without even penetrating the cell, inducing hyperproduction of inflammatory cytokines (cytokine storm), accumulation of angiotensin-II (ATII), and other humoral factors such as VEGF.

Excessive accumulation of inflammatory cytokines (IL-1β, TNF, IL-6, etc.), which are produced mainly by monocytes and macrophages, is characteristic of the severe course of COVID-19 (Merad and Martin, 2020). The maturation of IL-1β driven by the NLRP3 inflammasome depends on the formation of reactive oxygen species (ROS) in mitochondria of macrophages (Zhou et al., 2011). Leukocytes (especially neutrophils) produce significant amounts of ROS, causing additional endothelial dysfunction during inflammation. As we have recently shown, ROS produced by mitochondria (mtROS) play a key role in the activation of neutrophils by inducing the assembly and activation of NADPH oxidase (NOX2), which is responsible for the massive production of ROS (“oxidative burst”) in neutrophils (Vorobjeva et al., 2017, 2020). The same cross-talk between mtROS and NADPH oxidase has been described in endothelial cells (Nazarewicz et al., 2013).

Endothelial cells also produce the cytokines, and mitochondria are involved in the regulation of cytokine production. Induction of IL-6 expression with TNF is determined by the transcription factor NF-κB and also depends on mtROS (Zinovkin et al., 2014). In response to cytokines, adhesion molecules (ICAM1, VCAM1, E-selectin) are expressed on the endothelial surface, which promote adhesion and penetration of leukocytes through the vessel wall in tissue. The expression of these molecules is also dependent on NF-κB and is controlled by mtROS (Zinovkin et al., 2014; Romaschenko et al., 2015; Zakharova et al., 2017) (Figure 1).

Forrester et al. (2020) demonstrated that Drp1-dependent mitochondrial fragmentation induced by TNF in endothelial cells is critical for increasing mtROS, stimulating NF-κB-dependent expression of adhesion molecules, leukocyte adhesion, and pro-inflammatory proteomic changes. Suppression of Drp1 has been shown to prevent inflammatory activation of the endothelium both *in vitro* and *in vivo* in mesenteric postcapillary venules. Forrester et al. demonstrated that the canonical NF-κB signaling is responsible for TNF-dependent mitochondrial fragmentation. An additional mechanism of positive feedback can be mediated by mtROS, which activate both NF-κB (Zinovkin et al., 2014; Romaschenko et al., 2015; Zakharova et al., 2017) and NF-κB-independent fragmentation of mitochondria (Pletjushkina et al., 2006).

Inflammatory cytokines cause increased endothelial permeability to macromolecules, which may lead to pulmonary edema. TNF can decrease the expression of VE-cadherin, as well as activate proteolytic cleavage (shedding) of the extracellular domains of this protein, which determines the formation of adhesive intercellular contacts and isolating properties of microvascular endothelium. Besides, TNF induces a rearrangement of the actin cytoskeleton, phosphorylation of adhesion contact proteins, and activation of caspases, which enhances contact disassembly. Our experiments with the mitochondria-targeted antioxidant (MTA) SkQ1 showed that mtROS largely determines the effects of TNF in the endothelium (Zinovkin et al., 2014; Romaschenko et al., 2015; Galkin et al., 2016; Zakharova et al., 2017). It was shown that SkQ1 at nanomolar concentrations prevents an increase in the expression of adhesion molecules, shedding of VE-cadherin by matrix metalloprotease-9 (MMP9), rearrangement of the actin cytoskeleton, increase in endothelial permeability, and apoptosis.

The expression of IL-6, which plays an important role in the pathogenesis of COVID-19, is increased by TNF. In the acute phase of inflammation, these two cytokines act cooperatively stimulating endothelial activation and an increase in endothelial permeability. Signaling pathways activated by IL-6 in endothelial cells depend on STAT3 activation by Janus kinase, Notch signaling, and mitogen-activated protein kinases (MAPK) cascade, and are associated with an increase in mtROS, and activation of NADPH oxidase (Valle et al., 2019).

Another factor that probably plays an important role in pulmonary endothelial dysfunction during COVID-19 is vascular endothelial growth factor (VEGF). VEGF stimulates blood vessel growth, increases blood vessel permeability, and adhesion of platelets and leukocytes. Increased VEGF expression correlates with the severity of COVID-19 (Chi et al., 2020) and is known to be mediated by mtROS under inflammatory and hypoxic conditions (Hamanaka and Chandel, 2009). In turn, VEGF stimulates the production of mtROS and the activation of NADPH oxidases, which are critical for signal transduction (Kim et al., 2017).

The pathogenesis of COVID-19 is possibly associated with the local accumulation of the peptide hormone angiotensin-II (ATII) in the pulmonary capillaries. The accumulation of ATII can occur due to blocking the activity of the ACE2 protease that cleaves it and serves as a receptor for SARS-CoV-2 for cell entry. Signaling from the main ATII receptor (AT1) in the endothelium activates the MAPK cascade, as well as the transcription factor NF-κB, which leads to the expression of cytokines and adhesion molecules, as well as NO synthase (iNOS) and cyclooxygenase (COX2). Also, ATII activates NADPH oxidase (NOX2), which is the main source of ROS in the endothelium. Stimulation of endothelium with ATII was found to induce increased production of mtROS, which is necessary for the activation of both NF-κB and NOX2 (Nazarewicz et al., 2013; Itani et al., 2016).

### Mitochondrial ROS as a Promising Therapeutic Target

The data presented show that increased production of mtROS plays an important role in inflammatory activation and damage of endothelium in COVID-19 and may serve as a promising therapeutic target (Figure 1). Over the past two decades, several MTAs have been developed and tested. Mitochondrial-targeted ubiquinol (MitoQ) protected endothelial cells from oxidative stress (Dhanasekaran et al., 2004) and inhibited leukocyte-endothelial interactions (Escribano-Lopez et al., 2016). We have shown that mitochondria-targeted plastoquinol (SkQ1) prevents inflammatory endothelial dysfunction both *in vitro* and *in vivo*, as well as the lethal effect of TNF in a mouse model of the systemic inflammatory syndrome (Zinovkin et al., 2014; Romaschenko et al., 2015; Zakharova et al., 2017).

Another approach to preventing excessive inflammatory endothelial activation is based on the partial (or “mild”) uncoupling of oxidative phosphorylation, a therapeutic strategy proposed against pathologies associated with excessive production of mtROS, including aging (Cunha et al., 2011). However, the narrow therapeutic window for the dosage of uncouplers and the high toxicity do not allow their use in clinics. It was found that lipophilic cations such as dodecyltriphenylphosphonium (C_12_TPP) uncouple oxidative phosphorylation due to stimulation of the transmembrane cycling of endogenous free fatty acids coupled with proton translocation, while a decrease in membrane potential reduces their accumulation in mitochondria and thereby their uncoupling activity (Severin et al., 2010). This mechanism of self-limiting uncoupling may allow the use of these cations for therapy in a wide range of concentrations.

It has been shown that C_12_TPP inhibits endothelial activation *in vitro* and protects mice from TNF-induced systemic inflammatory syndrome (Romaschenko et al., 2015; Zakharova et al., 2017). It has been suggested that this effect is mediated by the antioxidant effect of mild uncoupling, which is explained by the fact that the maximum mtROS production occurs at the highest values of the membrane potential (Korshunov et al., 1997). Additionally, the protective effect of uncouplers may be associated with the activation of autophagy of dysfunctional mitochondria (mitophagy) (Lyamzaev et al., 2018). It is important to note that mild uncoupling reduces mitochondrial fragmentation (Romaschenko et al., 2015), which appears to be a critical event in the inflammatory activation of endothelial cells (Forrester et al., 2020). SkQ1, which is composed of the antioxidant residue plastoquinone conjugated to decyltriphenylphosphonium, also induces mild uncoupling (Severin et al., 2010), so the two mechanisms of SkQ1 action may jointly contribute to its anti-inflammatory effect.

Excessive production of mtROS in the endothelium (Chi et al., 2020) as well as in the neutrophils (Vorobjeva et al., 2017) depends on the opening of the mitochondrial permeability transition pore (mPTP). In turn, the pore opening can be stimulated by mtROS (Costantini et al., 1996). Inhibitors of mPTP that can block this vicious circle are currently being developed and studied as potential drugs for cardiovascular diseases (Briston et al., 2019). It is possible that these drugs, when combined with MTA or uncouplers, could be effective in preventing COVID-19 endothelial damage.

## Author Contributions

All authors listed have made a substantial, direct and intellectual contribution to the work, and approved it for publication.

## Conflict of Interest

The authors declare that the research was conducted in the absence of any commercial or financial relationships that could be construed as a potential conflict of interest.
